# Editorial for the Special Issue on “10th Anniversary of Nanomaterials—Recent Advances in Nanocomposite Thin Films and 2D Materials”

**DOI:** 10.3390/nano11082069

**Published:** 2021-08-15

**Authors:** Jordi Sort, Gemma Rius

**Affiliations:** 1 Departament de Física, Universitat Autònoma de Barcelona (UAB), E-08193 Cerdanyola Del Vallès, Spain; 2 Institució Catalana de Recerca i Estudis Avançats (ICREA), Pg. Lluís Companys 23, E-08010 Barcelona, Spain; 3 Instituto de Microelectrónica de Barcelona (IMB-CNM, CSIC), Campus UAB, E-08193 Cerdanyola del Vallès, Spain; gemma.rius@csic.es

As a way to celebrate the 10th anniversary of the journal Nanomaterials, this Special Issue within the section ‘Nanocomposite thin film and 2D materials’ provides an overview of the wide spectrum of research challenges and applications in the field, represented by a collection of 12 contributions, including three up-to-date review articles plus nine original works, in different targeted topics as described below.

The importance of synthesis and processing in the properties of compound or alloy thin films and their applications as functional coatings is reported in three of the contributed articles. Such is the case for perovskite BiFeO_3_ films presented by Micard et al. [1]). They use metal organic chemical vapor deposition (MOCVD) to optimize polycrystalline, pure phase thin films, as understood from XRD, EDX, and FE-SEM characterizations. Films piezoelectricity and ferroelectric property is confirmed by piezo force microscopy and spectroscopy, which envisions their use as lead-free hybrid energy harvesters. Another synthesis work is presented by Sudiyarmanto and Kondoh in [2]. In this case, supercritical fluid chemical deposition is applied to the production of Ni-Pt alloy thin films. By tuning the deposition rate with the precursors ratio, they obtain, as well, single-phase, polycrystalline material of the Ni-Pt alloy. The films are intended to be used as model catalyst surfaces due to their high activity and stability. In [3], the relevance of amorphous films is exemplified in contribution of Yan et al., in which a processing development on Mg-based metallic glasses for improving the corrosion resistance is presented. Particularly, first, MgO nanoplate arrays are coated by cyclic voltammetry treatments, and then, stearic acid is efficiently adhered to their surface, so that the corrosion resistance to NaCl solution is increased.

The versatility and flexibility of the growing and continuously evolving family of 2D materials is also well represented in this Special Issue. The advances in graphene materials are still generating new knowledge while still requiring significant technological progress. Novel methods to obtain graphene–polymer composite films with multiple functionalities, or their use for sensing platforms are two examples.

Van der Schueren et al. [4] show how co-mixing aqueous colloids plus casting of PVA and few-layer graphene can be used to obtain composite films with multiple functional properties. As their exfoliation method provided relatively large graphene flakes, the PVA-FLG composite exhibits good mechanical and electrical conductivity characteristics, as well as potential as an O_2_ barrier membrane based on a transmission rate reduction of 60%. In another contributed paper [5], graphene is used to realize interference-enhanced Raman scattering. Here, the key is to combine single-layer graphene with an ultra-thin alumina film on top of a metallic aluminum support. Correlating both experimental and theoretical results, their interference amplification can also be implemented. With this platform, on the basis of more conventional SERS, good results were obtained by simply adding ultra-small silver particles.

Closely related to the relevant and relatively new graphene and composites investigations domain, families of emerging materials such as 2D carbides and nitrides (MXene) and transistion metal dichalcogenides (TMD) are also receiving lots of attention and are thus represented in this Special Issue. The paper by Raagulan et al. [6] reviews reported works on MXene–graphene aerogel composites, with the focus on their use as electromagnetic interference shielding materials. Their efficiency is correlated with the obtained morpho-structural characteristics, while they compile relevant information in terms of processing techniques. In addition, centered on the specific functionalities of MXene materials, another review is provided by Ibrahim et al. [7] on their application for supercapacitors. In particular, they summarize the current knowledge and assess the progress in the self-standing MXenes as understood from their mechanical properties analysis and comparison to hybrid MXenes or other 2D materials.

Finding energy solutions in nanomaterials and, particularly, exploiting higher efficiencies derived from intrinsically high and, for instance, electrochemically-active, surface area of 2D materials has been an intense quest in the field. The original work provided by Hussain et al. [8] is a good example of the opportunities to investigate combinations of emerging materials and new synthetic processes. They develop a simple, one-pot, chemical reaction to produce hybrid W_2_C/WS_2_ nanostructured electrodes, which show good electrochemical performances for energy applications, such as hydrogen evolution and supercapacitors.

Another demonstration of the new developments needed for nanostructured, emergent 2D materials is given by Li et al. in [9]. In this case, they aim to facilitate integration toward industry by providing automated software methods and hyperspectral imaging hardware for characterizing MoS_2_ materials. Their convolutional neural-network-based algorithms demonstrated identification capabilities with a special resolution down to 100 nm and reasonable acquisition times in relatively large images.

Concluding, nanomaterials combinations including structural carbon films have a long run and will be the object of many research studies and developments in view of their commercial adoption. Versatile, abundant carbon, engineered at the nanoscale, still brings advances such as reflected in the following three papers, two of them about original developments of functional films or coatings and a concise review on RRAM devices.

Zhang et al. [10] combine carbon nitride films with TiO_2_ to obtain visible light enhanced photocatalysis, where the key of its efficiency comes from the optimization of plasma processing gases, while Sharma et al. [11] also use ion-based methods and study the synthesis of Li-C nanocomposites to evaluate their material potential for alternative batteries, such as based on Li–air interfaces. A comprehensive review on RRAM resistive switching mechanisms, materials, and bionic synaptic application is provided by Shen et al. [12] as a good example of the central role of nanomaterials in enabling sustainable and efficient solutions to current and future demands of our society.
